# Stable pH Suppresses Defense Signaling and is the Key to Enhance *Agrobacterium*-Mediated Transient Expression in *Arabidopsis* Seedlings

**DOI:** 10.1038/s41598-018-34949-9

**Published:** 2018-11-20

**Authors:** Yi-Chieh Wang, Manda Yu, Po-Yuan Shih, Hung-Yi Wu, Erh-Min Lai

**Affiliations:** 1 Institute of Plant and Microbial Biology, Academia Sinica, Taipei, Taiwan; 2 Molecular and Biological Agricultural Sciences Program, Taiwan International Graduate Program, Academia Sinica, Taipei, Taiwan; 3 Graduate Institute of Biotechnology, National Chung-Hsing University, Taichung, Taiwan; 4 Department of Plant Pathology and Microbiology, National Taiwan University, Taipei, Taiwan; 5 Biotechnology Center, National Chung-Hsing University, Taichung, Taiwan

## Abstract

*Agrobacterium*-mediated transient expression is a powerful analysis platform for diverse plant gene functional studies, but the mechanisms regulating the expression or transformation levels are poorly studied. Previously, we developed a highly efficient and robust *Agrobacterium*-mediated transient expression system, named AGROBEST, for *Arabidopsis* seedlings. In this study, we found that AGROBEST could promote the growth of agrobacteria as well as inhibit the host immunity response. When the factor of agrobacterial growth is minimized, maintaining pH at 5.5 with MES buffer was the key to achieving optimal transient expression efficiency. The expression of plant immunity marker genes, *FRK1* and *NHL10*, was suppressed in the pH-buffered medium as compared with non-buffered conditions in Col-0 and an *efr-1* mutant lacking the immunity receptor EFR recognizing EF-Tu, a potent pathogen- or microbe-associated molecular pattern (PAMP or MAMP) of *A*. *tumefaciens*. Notably, such immune suppression could also occur in *Arabidopsis* seedlings without *Agrobacterium* infection. Furthermore, the PAMP-triggered influx of calcium ions was compromised in the pH-buffered medium. We propose that the enhanced transient expression efficiency by stable pH was due to inhibiting calcium ion uptake and subsequently led to suppressing immunity against *Agrobacterium*.

## Introduction

*Agrobacterium tumefaciens* is a soil phytopathogen that naturally infects plant wound sites and causes crown gall disease in a wide range of plants, eudicot angiosperms and gymnosperms. Tumorigenesis is initiated from transfer of a piece of DNA, the transfer DNA (T-DNA) residing in the tumor-inducing (Ti) plasmid of *A*. *tumefaciens*, via a protein export machinery named the type IV secretion system from agrobacterial cells into the plant nuclear genome^1–4^. A disarmed but transfer-competent *A*. *tumefaciens* strain^5,6^ for *Agrobacterium*-mediated transformation of plants was generated by the removal of oncogenes in the T-DNA region residing in the Ti plasmid. This is a powerful tool for engineering various plant species to obtain desirable phenotypes for basic research and agriculture. By manipulating the infection condition, *A*. *tumefaciens* is able to genetically transform hosts beyond its natural hosts, including numerous dicot and some monocot plant species as well as other non-plant organisms such as fungi^7,8^, human cells^9^ and bacteria^10^. However, the transformation efficiency is highly variable among different species^11,12^ and efforts have been made to generate some universal protocols^1^ that can be easily adaptable in different laboratories to satisfy the need of plant transformation in different species.

*A*. *tumefaciens* can sense various plant-released signals to induce various chromosomal virulence genes (*chv*) or Ti-plasmid encoded virulence (*vir*) genes^13,14^. In conjunction with ChvE sugar-binding protein, the VirA-VirG two-component system can induce the expression of *vir* genes upon sensing phenolics, sugar, and acidity, the signals produced at plant wounding site. The transcription of *virG* gene highly depends on acidic pH via the ExoR-ChvG-ChvI regulatory cascade, which is also responsible for various acid-induced genes^14,15^. Therefore, application of acetosyringone (AS), a potent phenolics, to acidic minimal medium or buffer before *A*. *tumefaciens* co-inoculation with plants is a common practice to yield a higher transformation rate^16^. Surfactants such as Silwet L-77 and detergent Triton X-100 are also used to improve the cuticular penetration of fluid on the leaf surface and can significantly enhance the efficiency^17^.

The optimization of *Agrobacterium*-mediated transformation is mainly based on trial-and-error approaches, but the theoretical explanation is seldom addressed. A major breakthrough was the identification of the immune receptor in *A*. *thaliana*, which revealed the key molecular interaction between *A*. *tumefaciens* and *A*. *thaliana*. *A*. *thaliana* triggers its immune responses via recognizing elongation factor-thermo unstable (EF-Tu) of *A*. *tumefaciens* as a pathogen- or microbe-associated molecular pattern (PAMP or MAMP) via EF-Tu receptor (EFR); however, the elevated immune responses also hinder the transformation efficiency^18^. A mutant with loss of *EFR* function (i.e., *efr-1*) shows a significant increase in transient transformation efficiency and hence is used as a tool for rapid analysis of gene function or promoter activity^19^. However, the mutant background of *efr-1* may not be suitable for generating new mutants or for defence-related studies. This limitation was overcome in part by the development of a highly efficient transient transformation method known as *Agrobacterium*-mediated enhanced seedling transformation (AGROBEST), which optimizes the pH-buffered medium by supplementation with AB salts and glucose^19^.

Use of the *Agrobacterium*-mediated transiently expressed β-glucuronidase (GUS) reporter as an assay system significantly increased the transient transformation efficiency of wild-type Col-0 and *efr-1* mutant seedlings. The advantage of the method is to enhance the transient expression efficiency regardless of the genetic background of *A*. *thaliana*. In our previous study^19^, we showed an obvious enhancement in transient expression efficiency of *efr-1* seedlings over two other reported methods (FAST^20^ and Marion *et al*.^21^). Thus, the conditions optimized in the AGROBEST method may be able to suppress the residual defence mechanism on top of EFR-mediated resistance in *A*. *thaliana*. This residual defence mechanism could be rather primitive and essential for *A*. *thaliana* to protect against pathogens when a specific receptor for pathogen recognition (e.g., EFR for EF-Tu and FLS2 for flagellin) is not available in the plant species. However, the AGROBEST condition may also make *A*. *tumefaciens* more effective to infect *A*. *thaliana* independent of the host defence mechanism.

Here, we aimed to unveil the rationale underlying why AGROBEST can significantly enhance the transient expression/transformation efficiency in *A*. *thaliana* seedlings. We found that although the AGROBEST condition can increase the growth of *A*. *tumefaciens* during co-inoculation with *A*. *thaliana*, the main reason for the enhanced transient expression efficiency is stable acidic pH at 5.5. Further analysis showed that the stable pH was able to suppress the *A*. *thaliana* defence signalling pathway with or without *Agrobacterium* infection. Also, we provide evidence that the suppression of immunity could be due to ineffective calcium ion influx in the stable pH environment. The mechanistic insights gained from this study advance our understanding of *Agrobacterium–*plant interactions and provide invaluable information when using AGROBEST for dissecting gene function and regulatory pathways.

## Results

### AGROBEST promotes *Agrobacterium* growth and attachment to both Col-0 and *efr-1* seedlings

**Figure 1:**
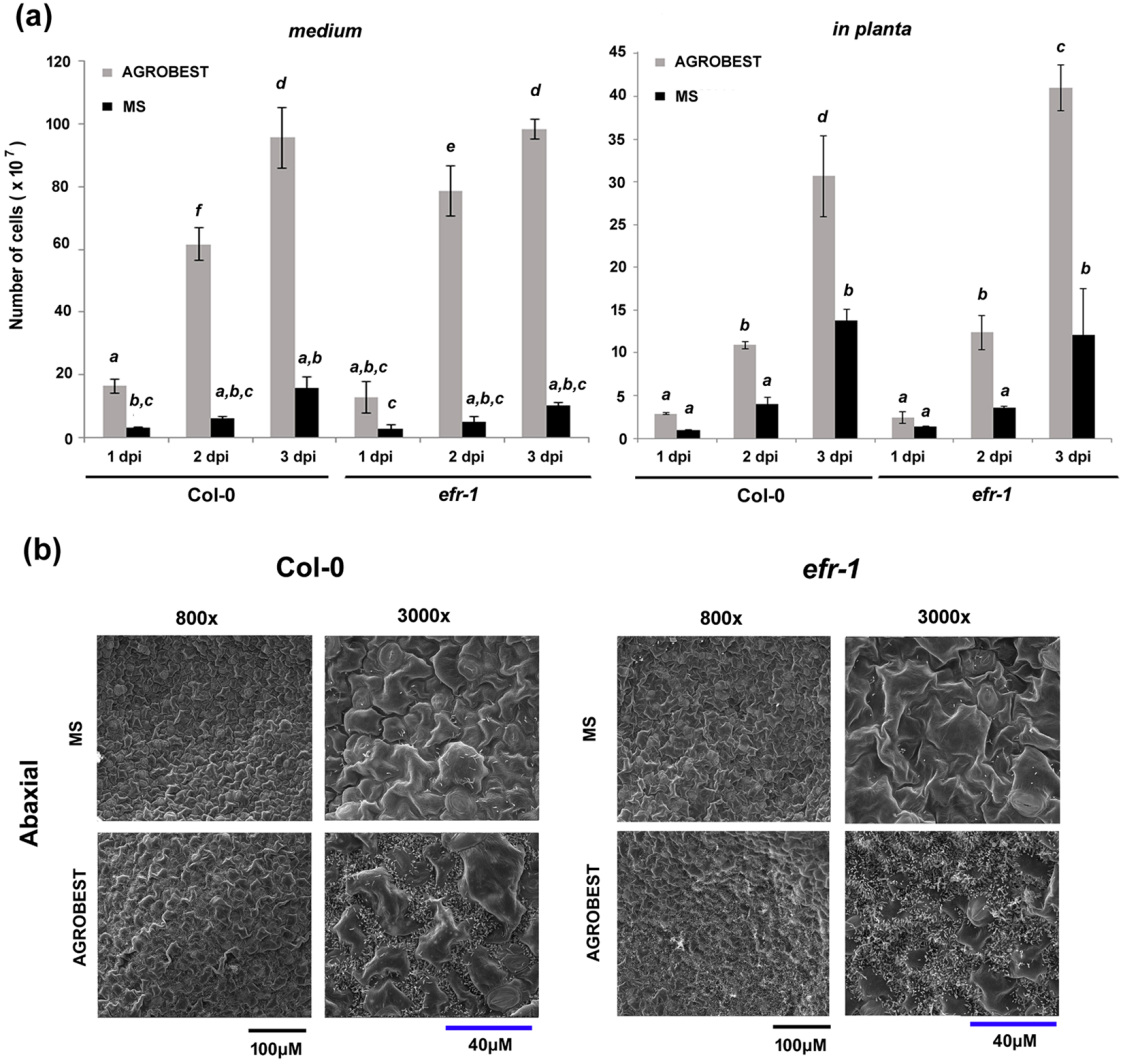
AGROBEST promotes *Agrobacterium* cell growth and attachment to Col-0 and *efr-1* seedlings. *Arabidopsis* Col-0 and *efr-1* seedlings were infected with *A*. *tumefaciens* C58C1(pTiB6S3ΔT)^H^ (OD_600_ = 0.02) harbouring pBISN1. (**a**) Number of *Agrobacterium* cells in the medium or associated with the seedlings (*in planta*) with different medium used (MS or AGROBEST) and 1–3 days of *Agrobacterium* infection. Data are mean ± SD from three biological replicates (~10 seedlings per well, three wells). Data were compared by a One-way ANOVA at the *P* < 0.05 level [F(11,24) = 211.63, *P* < 0.0001](medium), [F(11,24) = 86.39, *P* < 0.0001] (*in planta*). Different letters above the bars indicate statistically different groups. (**b**) Representative scanning electron microscopy images showing the attached *Agrobacterium* cells on the cotyledon surface (abaxial) of Col-0 or *efr-1* seedlings with AGROBEST or MS medium after 2 days of *Agrobacterium* infection. An overview (800x magnifications) and a close-up view (3000x magnification) are provided for each condition. Similar results were obtained from at least two independent experiments with multiple seedlings.

AGROBEST enhancing transient expression efficiency may be due to increased virulence or growth of *Agrobacterium* cells or promoting a plant’s susceptibility to *Agrobacterium* infection. Thus, we first investigated whether the AGROBEST condition could enhance the growth of *Agrobacterium* cells during inoculation with *A*. *thaliana* seedlings. We compared the viable *Agrobacterium* cell number in the medium and that associated in seedlings under two conditions, regular MS medium and AGROBEST. Transient GUS expression was 10-fold higher with AGROBEST than MS medium^19^. The MS medium contains ½ MS salts and 0.5% sucrose, and pH was adjusted to 5.5 with KOH in the presence of 200 μM AS. The AGROBEST medium includes an equal amount of the above-mentioned MS medium and AB-MES medium supplemented with 200 μM AS. The key difference between these two media is the addition of AB salts, glucose, and 2-(N-morpholino)ethanesulfonic (MES) to maintain pH at 5.5. In Col-0 seedlings, *Agrobacterium* cell number in medium was greater with AGROBEST than MS medium as early as 1 day post-infection (dpi) and the growth and difference continued up to 3 dpi. In *efr-1* seedlings, *Agrobacterium* cell number in medium was also greater with AGROBEST than MS medium although the difference at 1 dpi is not statistically significant. Both Col-0 and *efr-1* seedlings had more *in planta Agrobacterium* cells with AGROBEST than MS medium at 2 dpi and 3 dpi (Fig. 1a). Therefore, the AGROBEST medium could promote the growth of *Agrobacterium* cells during co-inoculation in both Col-0 and *efr-1* seedlings. To our surprise, in general, transient GUS expression was 5- to 10-fold higher in the *efr-1* mutant than Col-0 under the AGROBEST or MS condition^19^, but we could not detect a conclusive difference in number of *Agrobacterium* viable cells from either medium or *in planta* between co-culture of Col-0 and *efr-1* seedlings (Fig. 1a). Similar results were obtained from at least two independent experiments with multiple biological replicates except the *in planta Agrobacterium* viable cells in *efr-1* mutant were sometimes a bit higher than Col-0 (Fig. 1a) or no statistically different.

Besides counting the *in planta* viable cells, we used scanning electron microscopy (SEM) to examine *Agrobacterium* cells attached to *Arabidopsis* seedlings. In general, for both Col-0 and *efr-1* seedlings, density of attached agrobacterial cells was lower in roots than cotyledons; more cells were consistently attached to the abaxial than adaxial side of cotyledons. Hence, we compared the MS and AGROBEST conditions for agrobacterial cells attached to the abaxial side of cotyledons of Col-0 and *efr-1* seedlings (Fig. 1b). For both Col-0 and *efr-1* seedlings, density of agrobacterial cells on seedlings was higher with AGROBEST than MS medium. Similar to viable cell counting results, *Agrobacterium* cell density on the cotyledon surface did not differ between *efr-1* and Col-0 seedlings despite some variations in different areas of the same cotyledon.

In summary, the AGROBEST condition promoted cell growth in the medium and increased the overall cell number associated with seedlings and the attached cells on the cotyledon surface. Despite the cell number available for transformation being significantly higher with AGROBEST than MS medium, the marked increase in number of attached cells on the cotyledon with AGROBEST medium is nonetheless an interesting phenomenon to investigate whether the medium can make the plant tissue more welcoming to the bacteria. Thus, we further investigated whether the AGROBEST medium could have any additional impact on enhancing transient expression or transformation efficiency in addition to increased cell number in the inoculum.

### Stable pH at 5.5 is optimal for Col-0 and *efr-1* seedling transformation

**Figure 2:**
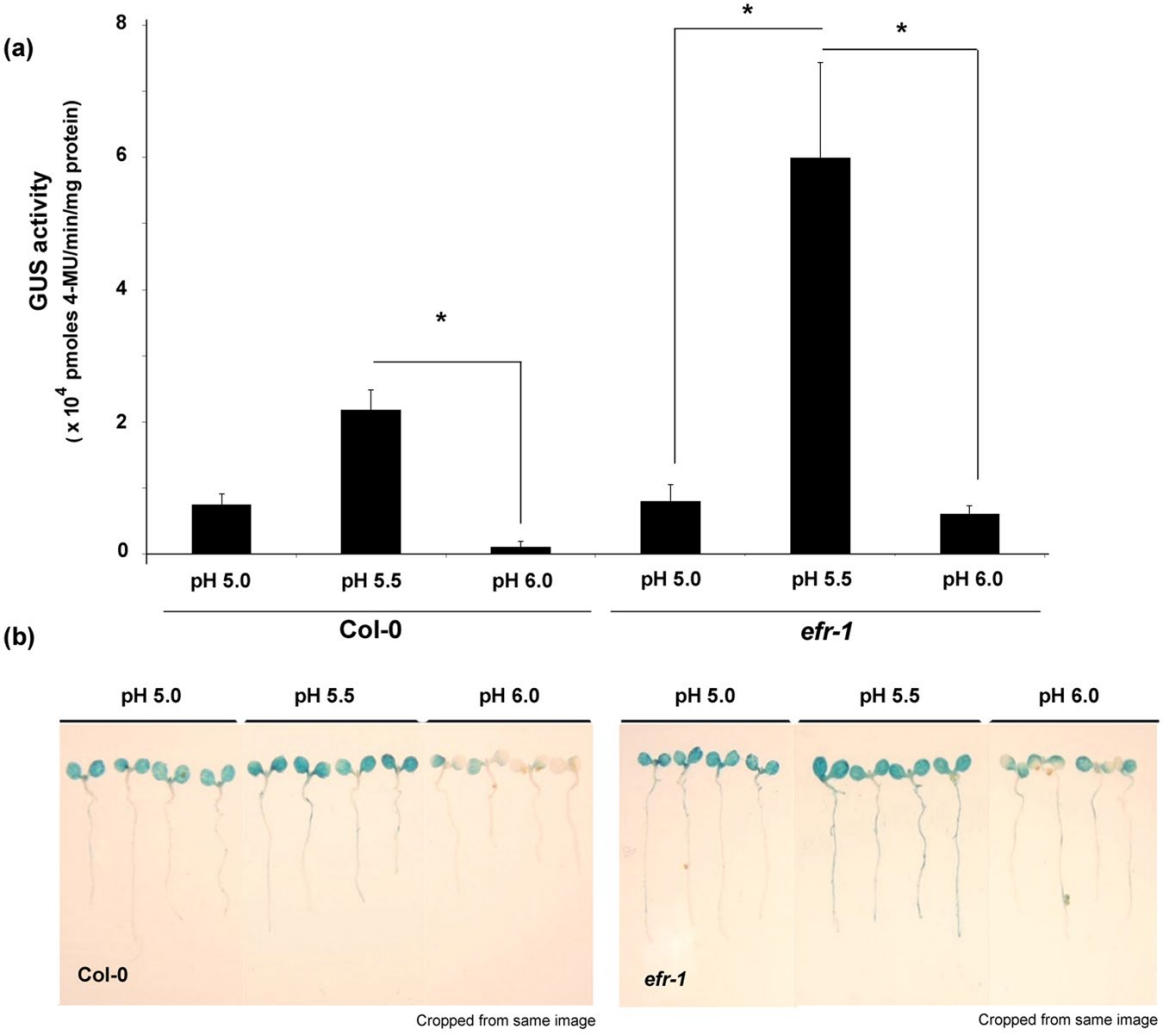
pH 5.5 is the optimal pH for transient GUS expression of Col-0 and *efr-1* seedlings. *Arabidopsis* Col-0 and *efr-1* seedlings were infected with *A*. *tumefaciens* C58C1(pTiB6S3ΔT)^H^ (OD_600_ = 0.02) harbouring pBISN1 in AGROBEST medium with buffered pH (5.0, 5.5 and 6.0) and were compared by quantitative GUS activity assay (**a**) and GUS staining (**b**) at 3 dpi. Data are mean ± SD GUS activity from three biological replicates (~10 seedlings per well, three wells). Similar results were obtained from three independent experiments. Significantly different values are denoted (*P < 0.05 by Student t test).

In our previous study^19^, we demonstrated that the AGROBEST condition is an optimal condition for *A*. *thaliana* seedling transient expression. As well, stable acidic pH at 5.5 buffered with MES or phosphate and AB salts in the co-culture medium are critical criteria in the optimization procedure. Thus, we next excluded the influence of medium composition such as AB salts on transformation efficiency and focused on the pH values to determine whether pH 5.5 is indeed optimal for transformation. The induction of *vir* genes by the phenolic compound AS can be activated at acidic pH 5.5-6.0^22^, so we used the defined AGROBEST medium buffered with MES to pH 5.0, 5.5, and 6.0 to test the effect of different acidic pH on transient expression efficiency. The highest transient GUS expression for both Col-0 and *efr-1* seedlings was at pH 5.5 (Fig. 2). The result for the best efficiency at pH 5.5 was expected, but we were surprised by the very low transient GUS expression at pH 6.0, the pH capable of efficient induction of *vir* gene expression *in vitro*^22^. Furthermore, we noticed greater enhancement for *efr-1* seedlings as compared with Col-0 at pH 5.5 versus pH 5.0 and 6.0. These results suggest that the massive increase in transient expression efficiency at pH 5.5 may not simply be explained by cell number in the inoculum nor activation of *vir* genes. Therefore, we designed the following experiments to study the role of stable pH 5.5 in seedlings under constant (e.g., inoculum cell density) and variable (e.g., with or without MES buffer) conditions.

### Stable acidic pH suppresses the defence response in *Arabidopsis* seedlings

**Figure 3:**
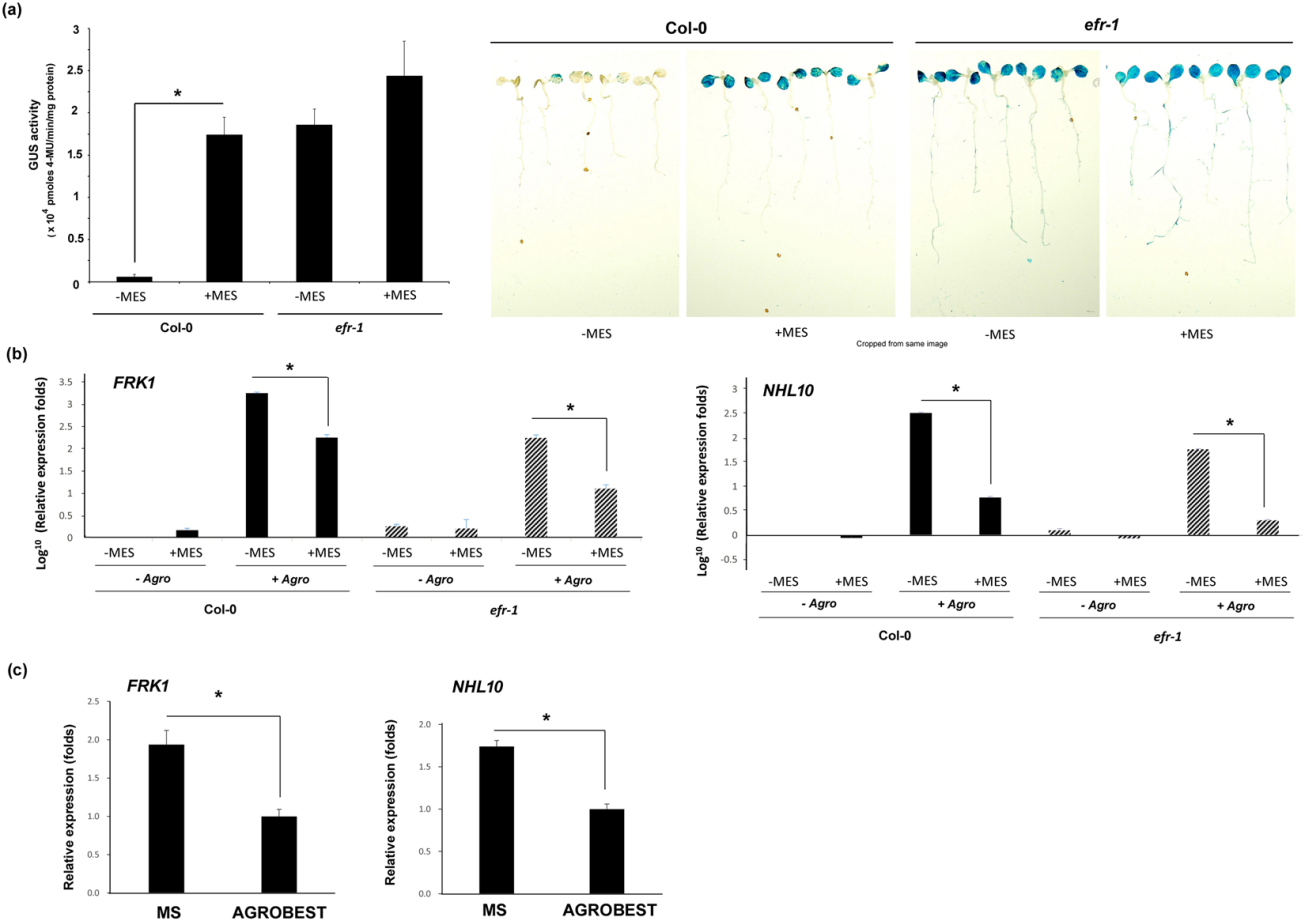
Stable pH 5.5 enhances transient expression and immune responses in *Arabidopsis* seedlings. (**a**) GUS activity and staining of Col-0 and *efr-1* seedlings infected with *A*. *tumefaciens* C58C1(pTiB6S3ΔT)^H^ (OD_600_ = 2) harbouring pBISN1 for 1 day in MS medium with or without MES followed by co-culture in fresh MS medium with timentin (100 µg/ml) for another 3 days. Data are mean ± SD GUS activity from three biological replicates (~10 seedlings per well, three wells). Similar results were obtained from three independent experiments. Expression of defence marker genes (*FRK1* & *NHL10*) (**b**) in Col-0 and *efr-1* seedlings with (+Agro) or without (−Agro) infection with *A*. *tumefaciens* C58C1(pTiB6S3ΔT)^H^ (OD_600_ = 2) harbouring pBISN1 for 1 day in MS medium with or without MES and (**c**) in Col-0 without co-inoculation of *A*. *tumefaciens* after 3 days in the medium with or without MES. qRT-PCR data are mean ± SD from three technical replicates. Similar results were obtained from three independent experiments. Significantly different values are denoted (*P < 0.05 by Student’s t test).

To study the effect of stable pH in *Arabidopsis* seedlings, we used a high cell density (OD_600_ = 2.0) of agrobacteria and shorter time (1 day) to minimize the growth difference in bacteria during inoculation. Because pH 5.5 is the optimal condition for transient transformation, we used the defined MS medium of adjusted pH 5.5 with or without MES buffer to test whether the transient expression efficiency was affected when the buffering system was absent. At fixed *Agrobacterium* cell density and inoculation conditions, transient GUS activity was strikingly increased in Col-0 seedlings grown in MES-buffered versus non-buffered medium (Fig. 3a). The data suggest that maintaining a stable pH to 5.5 is a key factor in achieving high transient expression/transformation efficiency. In addition, the GUS activity in Col-0 seedlings with MES medium was comparable to that in *efr-1* seedlings without MES, with still an effect for *efr-1* seedlings in MES on top of the –MES condition (Fig. 3a). This enhancement could be due to the suppression of the defence response by stable pH. Therefore, we detected the expression of some well-known defence marker genes: *FLG22-induced receptor-like kinase 1* (*FRK-1*) and *NDR1/HIN1-like 10* (*NHL10*). Both *FRK1* and *NHL10* are highly inducible upon contact with bacterial PAMPs^23^. The expression of *FRK1* and *NHL10* was highly induced in both Col-0 and *efr-1* seedlings after *Agrobacterium* infection (Fig. 3b). In both Col-0 and *efr-1* seedlings, *FRK1* expression at 1 dpi was significantly suppressed with MES medium, with suppression levels about 1 log_10_ (10-fold) lower with than without MES (Fig. 3b). Similarly, in both Col-0 and *efr-1* seedlings, the expression of *NHL10* at 1 dpi was suppressed more than 1.5 log_10_ (50-fold) with than without MES (Fig. 3b). Despite the significantly higher *Agrobacterium-*induced *FRK1* and *NHL10* expression in Col-0 than *efr-1*, the levels with MES suppressing the marker gene expression were approximately similar between Col-0 and *efr-1* seedlings. The ability of MES to suppress the defence gene expression in *efr-1* seedlings indicates that the suppression could be EFR-independent.

We also explored the possibility that the effect of suppression by MES was also observable when bacterial cells were absent. Although the expression was low in mock controls (4-day-old *Arabidopsis* seedlings grown in co-culture medium for 1 day without *Agrobacterium* infection), *FRK1 and NHL10* expression was also suppressed by MES (Fig. 3b). Similar suppression effects were observed in *Arabidopsis* seedlings grown in the original AGROBEST versus MS co-culture media for 3 days without *Agrobacterium* infection (Fig. 3c). Although not as significant as with *Agrobacterium* infection, the MES-mediated suppression effect on *FRK1* and *NHL10* expression was still observable, with about a 40% and 20% decrease, respectively, in seedlings without agrobacteria. The results confirm that maintaining stable acidic pH 5.5 is the key factor (adjustment) suppressing the defence response and leading to enhanced efficiency of *Agrobacterium*-mediated transient transformation of seedlings.

### Stable acidic pH inhibits the influx of calcium ion triggered by PAMPs

**Figure 4:**
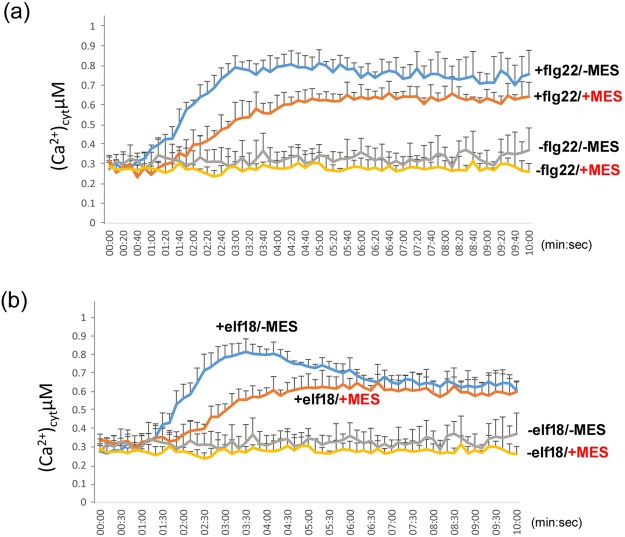
Stable pH 5.5 supresses calcium ion influx triggered by flg22 or elf18. The changes in cytosolic calcium (Ca^2+^) ion levels of *A*. *thaliana* Col-0 seedlings in MS medium with or without MES at pH 5.5 were monitored every 10 s for 10 mins. Application of flg22 (0.1 µM) or elf18 (0.1 µM) was at time = 0 by an automated injector. Data are mean ± SD from three biological replicates (~10 seedlings per well, three wells). Similar results were obtained in three independent experiments.

We speculated that maintaining a stable pH could lead to an imbalance in ion movement between the medium (or apoplasts) and cytosol, which undermines the normal defence signalling pathway. Calcium (Ca^2+^) ion plays important roles in defence signalling pathways^23–25^. Influx of Ca^2+^ ions from apoplasts into cytosol upon pathogen challenge is considered an important event to trigger downstream signalling pathways^26^. An increase in intracellular Ca^2+^ ion is sensed by some calcium-dependent protein kinases (CDPKs) and subsequently triggers the downstream defence pathways^23^. PAMPs such as flg22 and elf18 can induce Ca^2+^ influx from apoplasts into the intracellular space^27^. Thus, we compared cytosolic calcium levels upon PAMP induction in the medium with or without MES by using an aequorin-based method^28^. Aequorin is a 22-kDa protein that can bind calcium ions and a luciferin molecule, coelenterazine. Upon binding of calcium ions, aequorin converts coelenterazine to coelenteramide by its oxygenase activity. During the process, blue light at λ = 469 nm is emitted and detectable with a luminometer^29^. We used aequorin-expressing *A*. *thaliana* seedlings to detect the influx of calcium ions upon PAMP challenge. The main purpose of this experiment was to investigate any impact of calcium ion influx when pH was maintained at 5.5 with MES. When no PAMPs were added to the seedlings, cytosolic calcium ion level showed no increase (Fig. 4a,b), so seedlings were in a resting condition. In contrast, flg22 (Fig. 4a) or elf18 (Fig. 4b) could induce a prominent influx of calcium ions into the cytosol of *Arabidopsis* seedlings, which was consistent with previous observations^27^. Although an influx of calcium ions was observed in the sample with MES, the magnitude and the influx rate in MES-added samples were significantly lower than in the samples without MES. Calcium ion levels showed a steeper and higher increase in flg22-treated samples without MES (Fig. 4a). A maximum level, at about 0.8 µM, of cytosolic calcium ions was detected and it also appeared earlier, at about 3 min, as compared with about 4 min (0.6 µM) for samples with MES. A higher level was maintained until the end of the detection time (10 min) when MES was absent. Similarly, elf18 treatment (Fig. 4b) triggered a higher (~0.8 µM) and faster (~3:30 at maximum) influx of calcium ions without MES. A peak shape of the influx was observed only in samples without MES, whereas the levels decreased to similar levels for samples with MES at the end of the detection time (10 min). In addition, without PAMP treatment, the calcium ion levels were generally lower in seedlings with than without MES (Supplementary Figure S1). This observation may echo the reduced expression of defence marker genes in the presence of MES (Fig. 3b).

## Discussion

Our previous study showed that the highly enhanced transient expression efficiency of *efr-1* seedlings was achieved by two key factors, stable pH 5.5 and AB salts present in the AGROBEST medium^19^. Our current study found that the AGROBEST condition promoted *Agrobacterium* growth and attachment onto the seedling surface and suppressed the defence response. This situation may lead to enhanced transient GUS expression, an assay generally used to reflect transient transformation efficiency. We also extend the findings that buffered pH 5.5 is the key to this enhancement. The stable pH 5.5 suppressed the expression of the immune marker genes *FRK1* and *NHL10* independent of EFR, which could occur without *Agrobacterium* infection. Without MES to maintain pH at 5.5 during *Agrobacterium* infection, the pH value of the MS medium tended to fluctuate, often with up to 0.2 of pH increase after *Agrobacterium* infection. This finding is consistent with an alkalization effect upon triggering an immune response^18,30^ although we sometimes also observed decreased pH after *Agrobacterium* infection in non-buffered medium. Thus, we propose that the stable acidic pH could lead to a physical effect or a signal mimicking the absence of pathogens to trick plants to staying in a resting state with compromised immune response.

Although the number of *Agrobacterium* cells attached to the cotyledon surface of both Col-0 and *efr-1* seedlings was greater with AGROBEST than MS medium, transient transformation efficiency is not necessarily associated with a higher number of bound *Agrobacterium* cells. We previously reported a 5- to 10-fold higher transient GUS expression in the *efr-1* mutant than Col-0 under the AGROBEST or MS condition at 3 dpi with infection; the initial cell density was OD_600_ = 0.02^19^. With the same experimental setup in this study, both viable cell count and microscopy observations suggested no significant increase in number of plant-associated agrobacterial cells on *efr-1* seedlings versus Col-0 seedlings under the same condition (Fig. 1). This result is indeed consistent with previous findings of agroinfiltration in adult leaves revealing no higher *Agrobacterium* viable cells in leaves of the *efr-1* mutant, which is more susceptible to *Agrobacterium-*mediated transient GUS expression than Col-0^18^. Thus, EFR does not seem to play a major role in *Agrobacterium* attachment and rather acts on sensing the EF-Tu to trigger downstream immune responses in resistance to *Agrobacterium-*mediated transformation.

The suppression of the defence-response marker genes by MES indicates that the enhanced transient expression efficiency could be due to the seedlings in stable acidic pH being less capable of defence against *A*. *tumefaciens* and hence T-DNA more effectively transferred to the host. To this end, T-DNA–mediated transient GUS expression is a hallmark and standard assay to reflect *Agrobacterium-*mediated transient transformation efficiency^19–21,31^. However, we do not exclude that the increased GUS activity promoted by AGROBEST could also be due to increased T-DNA–encoded GUS gene expression besides increased copy number of T-DNA translocated from agrobacteria into the plant cells. Future work to develop a technically feasible tool quantifying the copy number of T-DNA in planta will shed light on the association of T-DNA copy number and transient gene expression levels inside the plant cells.

Uptake of calcium ions mainly relies on plasma membrane Ca^2+^/H^+^ exchangers, and export of H^+^/proton is a key event for the exchanger to function. Zhai *et al*.^32^ reported that the proton pump inhibitor vanadate could supress the activity of the Ca^2+^/H^+^ exchanger in the *Arabidopsis* membrane. In addition, optimal pH for the exchanger was 7.0, and acidic pH would reduce the efficiency of the exchanger^32^. We speculated that maintaining the pH at 5.5 with MES could reduce the activity of such Ca^2+^/H^+^ exchangers by inhibiting the proton movement across the plasma membrane. As the calcium ion influx is reduced, the upregulation of defence-response genes would be inhibited and thus the defence response would be weakened. The weakening of the defence response could make the *Agrobacterium* cells more effective for cell surface attachment as well as T-DNA transfer, which would lead to reduced transient expression or transformation efficiency. Because the effect of stable pH mainly targets the physical exchange of protons and calcium ions across the plasma membrane, the effect would be independent of many evolved defence mechanisms requiring molecular interactions. The enhanced transformation efficiency in *efr-1* seedlings supports that the immunity suppressed by stable pH is an extra mechanism that could be independent of the EFR signalling pathway. The activation of residual defence responses in *efr-1* seedlings may be via recognition of other PAMPs that rely on calcium signalling cascades such as CDPKs^23^. Thus, inhibition of Ca^2+^ influx is able to further suppress the defence response. Besides EF-Tu^30^, peptidoglycan and cold-shock protein but not flagellin of *Agrobacterium* can elicit plant immunity^33–35^. Practically, although knocking out *EFR* in *A*. *thaliana* can significantly enhance transient expression, the presence of other *Agrobacterium* PAMPs can still trigger the defence response to impede efficient *Agrobacterium*-mediated transformation, especially for some *Arabidopsis* ecotypes with unknown genetic background. Suppression of basal immunity via stable pH could be an alternative way to achieve satisfactory transient expression for various ecotypes. The mechanism of Ca^2+^ influx should be highly conserved across the plant kingdom. Thus, the findings in this study may be applied to increase both stable and transient transformation in other plant species recalcitrant to *Agrobacterium-*mediated transformation.

## Methods

### Materials and growth condition

*Arabidopsis thaliana* seeds of ecotype Columbia-0 (Col-0) and T-DNA insertion mutant *efr-1* (SALK_044334) were obtained from the Arabidopsis Biological Resource Center (Columbus, Ohio, USA). *A*. *thaliana* seedlings were grown in a growth room at 22 °C under a 16-hr/8-hr light–dark cycle (75 μmol m^−2^ s^−1^). *Agrobacterium tumefaciens* strain C58C1 (pTiB6S3ΔT)^H^^36^ harbouring pBISN1^37^ was used in the study for transient transformation assay. *A*. *tumefaciens* cells were routinely grown in 523 medium^38^ containing appropriate antibiotics with shaking (220 rpm) at 25 °C.

### *Agrobacterium* infection in Arabidopsis seedlings

The procedures for the seedling transient transformation assay were adapted from our previous report^19^ with modifications. The 523 grown *A*. *tumefaciens* cells were harvested and pre-induced with 200 μM acetosyringone in AB-MES (17 mM K_2_HPO_4_, 8 mM NaH_2_PO_4_, 18 mM NH_4_Cl, 2 mM KCl, 1.25 mM MgSO_4_, 100 µM CaCl_2_, 10 µM FeSO_4_, 50 mM MES, 2% glucose (w/v), pH 5.5) at 25 °C for 16 hr and resuspended in appropriate buffer with cell density OD_600_ = 0.02 or 2.0. *A*. *thaliana* seeds were germinated in regular MS medium (1/2 MS, 0.5% sucrose [w/v], pH 5.5) (~10 seeds in each well) and 4-day-old seedlings were then incubated in 1 ml respective co-culture medium in one well of 6-well plates for *Agrobacterium* infection up to 3 days (for infection at OD_600_ = 0.02) or 1 day (for infection at OD_600_ = 2.0) followed by the co-culture medium being replaced by fresh MS medium (1/2 MS, 0.5% sucrose [w/v], pH 5.5) with timentin (100 µg/ml) for another 3 days. The AGROBEST method used was described previously and the co-culture medium used was MS medium and AB-MES at a 1:1 ratio. The seedlings were then removed for GUS staining immediately or stored at −80 °C before RNA extraction or quantitative GUS activity assay.

### Plant RNA extraction and quantitative RT-PCR

Total RNA was extracted from *A*. *thaliana* seedlings by using the RNeasy Plant Mini Kit (Qiagen). First-strand cDNA was synthesized from 4 μg total RNA with SuperScript III Reverse Transcriptase (Invitrogen) and oligo (dT) primers. Quantitative PCR involved using the QuantStudio 12 K Flex Real Time PCR system (Applied Biosystems) with the Power SYBRR Green PCR Master Mix (Invitrogen). Primers for *A*. *thaliana FRK1* (At2g19190) and *NHL10* (At2g35980)^23^ were synthesized according to the sequences of a previous study. ACTIN 2 (At3g18780) was an internal control. Data were compared by Student t test from three technical replicates for each independent experiment.

### GUS staining and activity assay

Seedlings were stained with 5-bromo-4-chloro-3-indolyl glucuronide (X-Gluc) at 37 °C for 6 hr as described^39^. GUS activity assay was determined by the conversion of 4-methylumbelliferyl-β-D-glucuronide (4-MUG) to 4-methylumbelliferone (4-MU). 4-MU fluorescence (ex. 356 nm, em. 455 nm) was measured by using a 96 microtiter-plate reader (Bio-Tek Synergy Mx) and the specific GUS enzyme activity was calculated based on 4-MU standards. Data were compared by Student t test from three biological replicates for each independent experiment.

### *Agrobacterium* viable cell count and electron microscopy

Seedlings infected with *A*. *tumefaciens* were rinsed briefly with 0.9% saline. Seedling-associated *A*. *tumefaciens* cells were extracted by grinding the seedlings in 0.9% saline (1 ml for 10 seedlings from each well). The extracts or inoculation medium were serially diluted and spread on low-salt LB (0.5% NaCl) agar plates for incubation at 25°C for 2 days before counting colony-forming units (cfu). *Agrobacterium* cells on the seedling surface were observed by scanning electron microscopy (SEM, FEI Quanta 200). The samples were first mixed with fixation solution (2.5% glutaraldehyde, 4% paraformaldehyde in 0.1 M sodium phosphate buffer, pH 7.0) at room temperature overnight. The samples were rinsed with 0.1 M sodium phosphate buffer, pH 7.0 three times and post-fixed in 1% osmium tetroxide (OsO_4_) for 4 h then rinsed with 0.1 M sodium phosphate buffer, pH 7.0 three times^40^. Before viewing, samples were dehydrated in an ethanol series and dried with use of a Hitachi HCP-2 critical point dryer. SEM at 20KV was used for viewing.

### Cytosolic calcium ion measurement

Aequorin-expressing *Arabidopsis* seeds were a gift of Prof. Marc Knight (Durham University, Durham, UK). Measurement of cytosolic calcium ions was based on a published report^28^ with modifications. Ten-day old seedlings were first incubated in MS medium with or without MES for 1 day, transferred into 1 well of 12-well plates with 500 μl reacting solution (MS medium at pH 5.5 with 10 mM KCl, 10 mM CaCl_2_, 100 mM MgCl_2_ and 10 μM coelenterazine, + /− MES) and vacuum-infiltrated for 5 min, then incubated in the dark for 3 h. The same reacting solution (500 μl, with or without flg22 (0.2 μM)/elf18 (0.2 μM)) was loaded into the solution automatically in the reader (Bio-Tek Synergy Mx), and luminescence at λ = 469 nm was measured immediately at every 10 s for 10 min. Data were compared by Student’s t test from three biological replicates for each independent experiment.

## Electronic supplementary material

Supplementary Information
