# Supplementary material for: Stable pH Suppresses Defense Signaling and is the Key to Enhance Agrobacterium-Mediated Transient Expression in Arabidopsis Seedlings
Source: Sci Rep. 2018 Nov 20;8:17071. doi: 10.1038/s41598-018-34949-9 (PMC6244089; doi:10.1038/s41598-018-34949-9)
Supplement: Supplementary file 1 — Supplementary Information [file 41598_2018_34949_MOESM1_ESM.pdf]

Supplementary Information for

Stable pH Suppresses Defense Signaling and is the Key to Enhance *Agrobacterium*-Mediated Transient Expression in *Arabidopsis* Seedlings

Yi-Chieh Wang[1,+], Manda Yu[1,+], Po-Yuan Shih[1,2,3], Hung-Yi Wu[1,4], and Erh-Min Lai[1,2,3,4,5*]

[1]Institute of Plant and Microbial Biology, Academia Sinica, Taipei, Taiwan
[2]Molecular and Biological Agricultural Sciences Program, Taiwan International Graduate Program, Academia Sinica, Taipei, Taiwan
[3]Graduate Institute of Biotechnology, National Chung-Hsing University, Taichung, Taiwan
[4]Department of Plant Pathology and Microbiology, National Taiwan University, Taipei, Taiwan
[5]Biotechnology Center, National Chung-Hsing University, Taichung, Taiwan

[+]these authors contributed equally to this work

*Correspondence to emlai@gate.sinica.edu.tw

Supplementary Figure S1

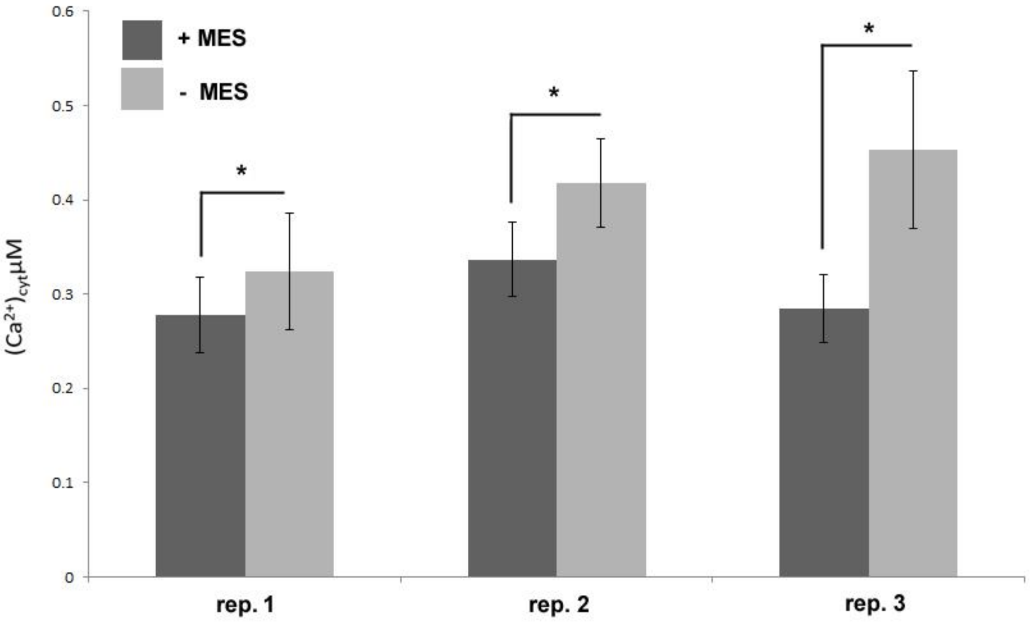

Supplementary Figure S1. Stable pH reduces basal cytosolic calcium levels. Cytosolic calcium (Ca^{2+}) ion levels in *A. thaliana* Col-0 seedlings in MS medium with or without MES at pH 5.5. No PAMPs were applied and seedlings were monitored every 10 s for 10 mins. Each bar represents the mean±SD of a total of 180 readings (60 x 3 replicates) and data were compared between seedlings in medium with or without MES by Student t test. Significantly different values are denoted (**P*<0.001). Data are from three independent experiments (rep.1, rep.2 and rep. 3).
